# Modeling the Developmental Patterns of Auditory Evoked Magnetic Fields in Children

**DOI:** 10.1371/journal.pone.0004811

**Published:** 2009-03-11

**Authors:** Rupesh Kotecha, Maria Pardos, Yingying Wang, Ting Wu, Paul Horn, David Brown, Douglas Rose, Ton deGrauw, Jing Xiang

**Affiliations:** 1 MEG Center, Department of Neurology, Cincinnati Children's Hospital Medical Center, Cincinnati, Ohio, United States of America; 2 Department of Mathematical Sciences, University of Cincinnati, Cincinnati, Ohio, United States of America; 3 Ear and Hearing Center, Cincinnati Children's Hospital Medical Center, Cincinnati, Ohio, United States of America; 4 Division of Audiology and Department of Pediatric Otolaryngology, Cincinnati Children's Hospital Medical Center, Cincinnati, Ohio, United States of America; Institut de la Vision, France

## Abstract

**Background:**

As magnetoencephalography (MEG) is of increasing utility in the assessment of deficits and development delays in brain disorders in pediatrics, it becomes imperative to fully understand the functional development of the brain in children.

**Methodology:**

The present study was designed to characterize the developmental patterns of auditory evoked magnetic responses with respect to age and gender. Sixty children and twenty adults were studied with a 275-channel MEG system.

**Conclusions:**

Three main responses were identified at approximately 46 ms (M50), 71 ms (M70) and 106 ms (M100) in latency for children. The latencies of M70 and M100 shortened with age in both hemispheres; the latency of M50 shortened with age only in the right hemisphere. Analysis of developmental lateralization patterns in children showed that the latency of the right hemispheric evoked responses shortened faster than the corresponding left hemispheric responses. The latency of M70 in the right hemisphere highly correlated to the age of the child. The amplitudes of the M70 responses increased with age and reached their peaks in children 12–14 years of age, after which they decreased with age. The source estimates for the M50 and M70 responses indicated that they were generated in different subareas in the Heschl's gyrus in children, while not localizable in adults. Furthermore, gender also affected developmental patterns. The latency of M70 in the right hemisphere was proposed to be an index of auditory development in children, the modeling equation is 85.72-1.240xAge (yrs). Our results demonstrate that there is a clear developmental pattern in the auditory cortex and underscore the importance of M50 and M70 in the developing brain.

## Introduction

The maturational changes and developmental abnormalities of the auditory system can be detected with magnetoencephalography (MEG) [1]. Neuromagnetic responses in the auditory cortex to an auditory stimulus, termed auditory evoked fields (AEFs) include several components. A component approximately 100 ms after stimulus presentation (or M100) has been considered the most prominent response in the auditory system in adults [2]. However, a recent report has demonstrated that it is troublesome to localize the auditory cortex using M100 [3]. An earlier component of the middle latency components of AEFs( or M50), is somewhat less studied, predominantly due to the fact that it tends to be smaller in amplitude and less reliably observed in adults [4], [5]. Interestingly, in children, M50 has been found to be larger than M100. Furthermore, intracerebral recordings have demonstrated that the M50 is probably a complex that includes two subcomponents: Pb1 and Pb2 [6]. It remains unclear; however, if and how the Pb1 and Pb2 subcomponents change in the developing brain.

The latency of M100 is dependent on age in healthy developing children, with prolonged latencies for children as young as 4 years old and shorter latencies for those approaching adulthood [7], [8]. This shortening of the auditory response latency is a reflection of typical neurophysiological maturational processes including synaptogensis, pruning, dendritic arborization, and myelination of thalamo-cortical and cortico-cortical projections [9]. Therefore, the latency of M100 may be an effective indicator of auditory function in developing children or provide indications of deficits in auditory processing [1]. The considerably less studied components of the auditory waveform are the earlier tone responses, often termed auditory evoked middle latency components (MLCs). It also should be clear that analysis of the electroencephalograph (EEG) counterparts of MLCs, so called the middle latency auditory evoked responses, has revealed two separate robust peaks appearing at about 52 ms and 74 ms after stimulus presentation [5]. When the results are combined with those from MEG, these early components of MCLs exhibit much smaller amplitude in adults compared to later auditory responses, such as M100. Alternatively, examinations of the AEF waveforms of children have revealed that these peaks are quite reproducible. In fact, EEG analysis has suggested the possible existence of a time-course of auditory developmental pattern in children the timing of this middle latency response [10].

To characterize the development of the auditory system in children, past studies primarily utilized EEG. As an alternative, MEG noninvasively measures cortical neuromagnetic activation in the auditory cortex with both a high spatial [11] and temporal resolution [12]. In comparison to EEG, these MEG characteristics could allow for the separation of the middle latency components (M50 and M70) [13]. Moreover, the possibility of volumetric localization has recently been developed for MEG and used to analyze specific regions of the brain [14], [15]. This could prove useful in determining the exact subareas in Heschl's gyrus from which auditory activity is generated. Furthermore, using MEG, it is possible to perform coherence analysis of cortical activity and investigate the high-frequency neuromagnetic signals of the temporal lobe [16]. For future investigations determining the differences in the high-frequency neuromagnetic signals occurring during these components, MEG is also well suited. Other similar functional imaging modalities, such as functional magnetic resonance imaging (fMRI) and positron emission topography (PET) do not have the temporal resolution to accurately capture these signals. Noticeably, because of these strengths, MEG tends to be very useful in both clinical practice and research. The study of different cognitive disorders and developmental disabilities are now well within the domain of clinical MEG use. For example, the absence of specific characteristics of the M50 and the M100 generators are known to correlate with child-onset schizophrenia [17], [18]. The study of developmental dyslexia is also well indicated using MEG by detecting abnormal hemispheric asymmetry patterns. Past research has shown that the absence of a normal response from the left hemisphere in the temporo-occipital area correlates closely with dyslexia [19]. Currently, MEG is being used to indicate improvement of children with dyslexia in reading and writing skills [20]. In addition to the study of these disorders, the most widespread utilization of MEG is in the identification of epileptic foci in pediatric epilepsy [21]. Therefore, clinically, MEG recordings are most useful in mapping critical functional regions, such as the auditory cortex, for brain surgery.

Despite interesting EEG findings of these early components of MLCs in many studies, only several studies have examined these components using MEG in children. Moreover, these relatively few studies have only identified one middle latency component, without separating the middle latency response into its distinct subcomponents as identified with EEG. One study revealed that the response (occurring at approximately 70 ms) exhibits a characteristically larger amplitude than the traditionally studied M100 in children [22]. Continuing this potentially valuable vein of research, Oram Cardy and colleagues conducted additional studies to further demonstrate that the latency of this early response is a useful indicator of language functioning and comprehension development in children [23]. However, the developmental patterns based on the individual subcomponents of the middle auditory responses in healthy children with MEG has yet to be empirically demonstrated. For example, previous MEG reports have demonstrated that the middle auditory evoked responses, M50 and M70, have been localized in the auditory cortices [4], [24], [25]. However, it remains unclear if M50 and M70 are generated by the same group of neurons or if their source locations change with age. From our point of view, it is necessary to systematically investigate the source locations of M50 and M70 in the developing brain since they cannot be identified easily in the matured brain.

The first objective of the present study was to determine if the middle latency auditory evoked components (M50 and M70) in children aged 6–17 year old vary in latency, amplitude and source locations. The secondary objective was to model the developmental patterns of M50, M70 and M100 in AEF. To explore the possibility of mathematically describing or predicting the maturational changes of the three components, we characterized the development patterns of the middle latency components in AEF with a computational model. We consider that the normal computational model may help to accurately identify developmental delays or auditory abnormalities in the pediatric population. Further analysis may also determine if the responses from the left and right hemispheres have the same significance in describing and predicting the developmental changes. To determine the most reliable age-dependent AEF components, the data obtained from children were compared to data recorded from adults with an identical stimulation paradigm. We hypothesized that a comprehensive analysis of M50, M70 and M100 would reveal at least one component that changes significantly with age. We further hypothesized this component would be mathematically modeled and used as an objective developmental index for the study of functional development of the brain.

## Results

**Figure 1:**
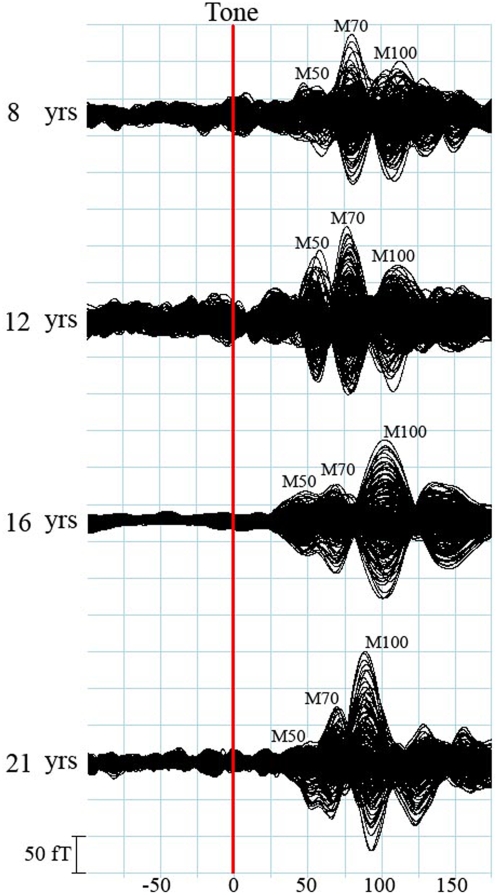
Representative auditory evoked fields (muti-channels) showing identifiable M50, M70 and M100 peaks in children from each of the age groups and one adult. The red line indicates the onset of the auditory stimulation (or trigger). Noticeably, the morphology, amplitude and latency of the three component changes with age significantly.

To analyze the left and right responses individually, the MEG channels were separated into two groups: the left group covered the left hemisphere and the right group covered the right hemisphere. The response identified in the left group was indicated with “L” (e.g. M50L); the response identified in the right group was indicated with “R” (e.g. M50R). The waveforms showed M50 responses in 42 children (42/60, 70%), M70 in 57 children (57/60, 95%), and M100 in 60 children (60/60,100%). As the focus of this study was to describe the developmental changes in children, and the number of adult subjects was small compared to the age range (30 years), analysis primarily focused on the 6–17 year age range. Moreover, preliminary analysis of the adult data showed no significant changes across the age range. Figure 1 shows typical waveforms with all three responses from an individual in each age group. We also noted components after M100; however, this study focused on the early components and did not analyze the later responses. This figure illustrates the changes in morphology that occur with increasing age in children. In general, the amplitude of M70 increased and then decreased with age, the amplitude of M100 became larger as age increased, and the latency for all waves decreased as age increased. Separation by gender did not yield any unique findings of amplitude or latency for these responses.

### Latency Characteristics

**Figure 2:**
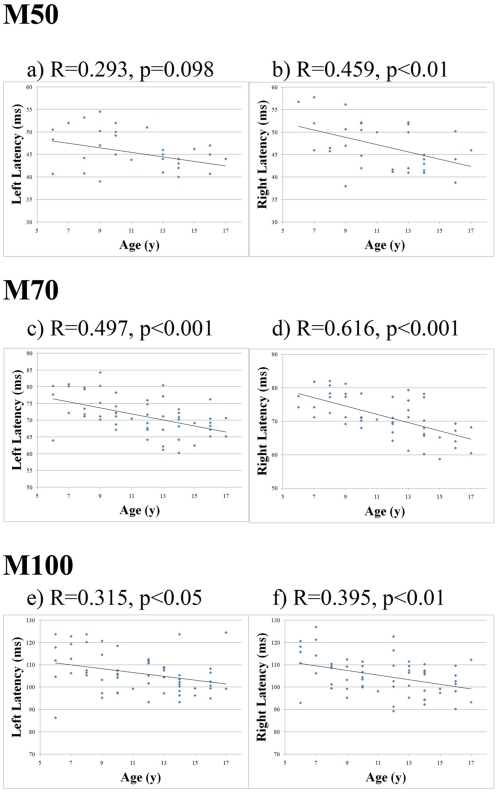
Scatter plot of the peak latencies of the M50, M70 and M100 components in the left and right auditory hemispheres relative to chronological age of the children. Linear regression lines, using the least-squares method, are also plotted to show general trends for each hemisphere. The M70 components show a clear developmental change with the highest correlation coefficient.

Across the three different groups of children, the latencies for the responses in both the right and left hemispheres decreased as age increased. As shown in Figure 2 and Table 1, the mean latency of each response decreased with age. Linear regression analysis showed that across the entire age range, M50R shortened with age (p<0.01). Comparison of the youngest group (6–9 years) and the oldest group (14–17 years) also showed that the older children had a significantly shorter M50R latency by 5.96 ms (p<0.05). Regression analysis of the latency of M50L with respect to age also showed a decreasing directional trend. This result was supported by a statistically significant difference in latency of 3.59 ms between the youngest group of children and the oldest group (p<0.05).

**Table 1:** Mean values (±SD) for latencies of M50, M70, and M100 auditory evoked responses to binaural tone stimulation.

Hemisphere	Age (years)	M50	M70	M100
		Latency (ms)	Latency (ms)	Latency (ms)
Left	6–9	47.28±5.16	75.75±5.39	110.52±10.54
	10–13	44.61±7.74	70.71±5.08	105.53±6.02
	14–17	43.69±2.28	68.23±4.05	102.97±8.37
Right	6–9	49.68±6.22	76.45±4.16	109.29±9.17
	10–13	47.03±4.87	71.26±4.71	104.48±7.83
	14–17	43.72±3.28	66.49±5.54	101.43±6.70

When considering the entire child age range, the latency of both M70L and M70R decreased with age (p<0.001 each). In fact, the latency of M70 in both right and left hemispheres showed the most significant differences between the groups of children. The M70L latency in the youngest children was significantly longer than the M70L latency in either the mid-aged group or the oldest children by 5.04 ms and 7.52 ms, respectively (p<0.01 and p<0.001, respectively). Correspondingly, the latency of M70R in the oldest children was considerably shorter than the youngest by 9.96 ms or mid-aged children by 5.19 ms (p<0.005 and p<0.001, respectively).

Linear regression analysis also showed that the M100L and M100R latency decreased with age (p<0.05 and p<0.01, respectively). Furthermore, group comparisons revealed these differences. For example, the youngest children showed significantly longer M100L and M100R latencies than the oldest children by 7.55 ms and 7.86 ms, respectively (p<0.05 and p<0.01, respectively). Comparisons of the relationship between component latency and age between hemispheres showed that the latency shortened faster in the right hemisphere than in the left. The latencies of all subjects are summarized in Table 1; linear regression analysis is presented in Figure 2.

### Amplitude Characteristics

**Figure 3:**
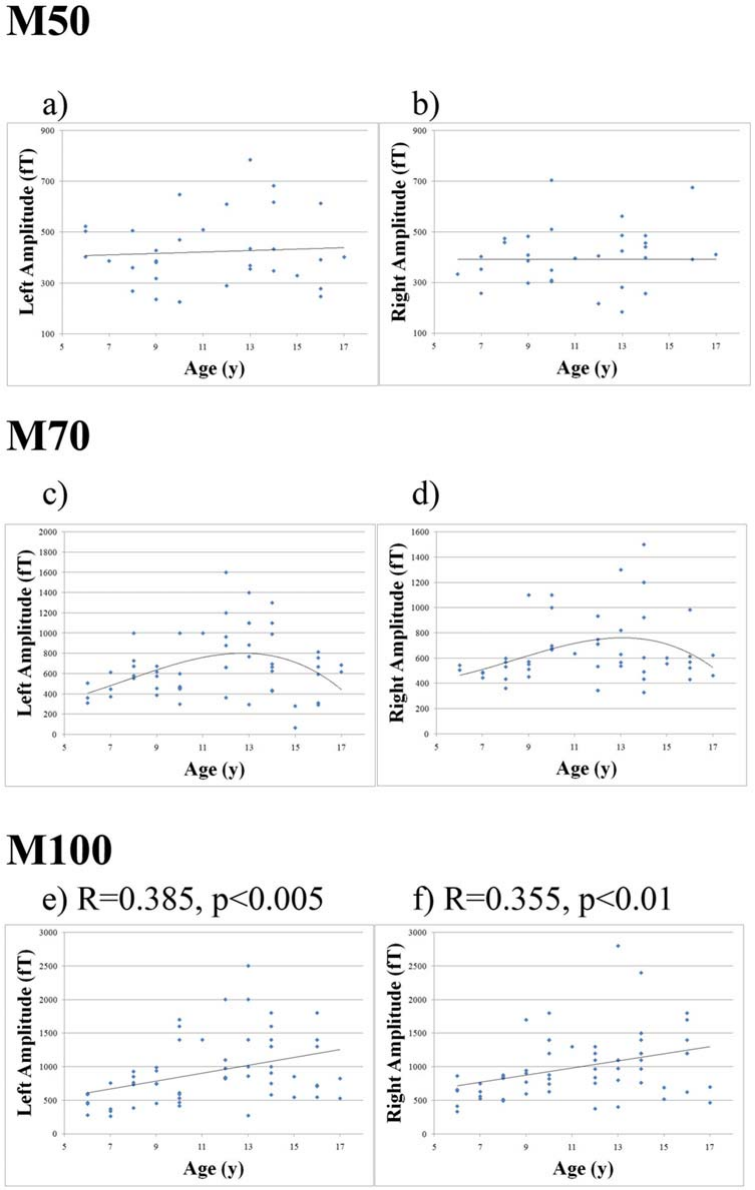
Scatter plot of the peak RMS amplitudes of the M50, M70 and M100 components in both auditory hemispheres relative to chronological age of the children. The plots of “left amplitude” represent the left hemisphere; the plots of “right amplitude” represent the right hemisphere. General trends are also plotted for each hemisphere. Noticeably, the amplitude of M70 does not change with age linearly. Instead, it reaches the peak around 12–14 years old and then decreases with age.

The amplitudes of M50L and M50R seemed to have no significant relationship with age as determined with linear regression analysis and possible complex fitting. Conversely, the amplitudes of both the M70L and M70R seemed to have a significant relationship with age. To investigate this relationship, a linear regression was first applied to the data. Alternatively, a more complex fit, as suggested from analysis of group comparisons, proved to be more appropriate (solid curve, Figure 3). The amplitudes of both M70L and M70R increased with age, until the child reached 13–14 years of age. For example, the M70L and M70R amplitudes were smaller in the youngest children by 237.69 fT and 176.77 fT than in the mid-aged group (p<.05 each). After the children reached 13–14 years of age, though, a negative correlation between M70L and M70R amplitude was observed.

Interestingly, the amplitudes of the M100L and M100R peaks increased linearly with age (p<0.005 and p<0.01, respectively). Comparisons between groups of children also revealed these differences. The M100L and M100R amplitudes were statistically smaller in the youngest children than in the middle aged children by 485.32 fT and 355.77 fT, respectively (p<0.005 and p<0.01, respectively). Moreover, the M100L and M100R amplitudes recorded in the youngest children were significantly smaller than the peak amplitudes in the oldest children by 432.89 fT and 444.91 fT, respectively (p<0.005 each). The amplitudes of all subjects are summarized in Table 2; corresponding graphs are presented in Figure 3.

**Table 2:** Mean values (±SD) for amplitudes of M50, M70, and M100 auditory evoked responses to binaural tone stimulation.

Hemisphere	Age (years)	M50	M70	M100
		Amplitude (fT)	Amplitude (fT)	Amplitude (fT)
Left	6–9	391.35±90.85	553.95±172.49	612.92±234.67
	10–13	447.20±180.02	791.64±366.76	1098.24±585.45
	14–17	434.00±152.43	629.51±306.27	1045.81±427.08
Right	6–9	385.36±75.41	543.19±199.30	730.68±293.84
	10–13	394.80±145.13	719.96±318.75	1086.45±509.03
	14–17	393.93±174.83	708.38±222.99	1175.59±493.93

### Source Localization

The M50L, M70L and M100L were localized to the left Heschl's gyrus and the M50R, M70R and M100R were localized to the right Heschl's gyrus. It was noted that the M70 response was much stronger and clearer than the M100 response 25% in children aged from 10–13 years old (16/20 of the children, 80%). It seemed that M70 was the appropriate component for mapping the auditory cortex for children in an age range of 10–13 years old.

**Figure 4:**
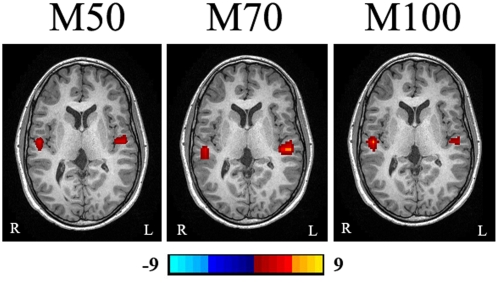
Magnetic Source Imaging (MSI) from mid-aged child shows that the M50, M70 and M100 AEF components are localized to subareas of Heschl's gyrus. The location of M50 is anterior to that of M70. The color bar shows the strength of neuromagnetic activation. The “L” indicates the left side and the “R” indicates the right side.

Wavelet-based beamformer results also showed that the M50 and M70 response peaks were localized to different subareas of the Heschl's gyrus. In the left hemisphere, the range for the median distance between the two sources was 12.1–58.5 points (a 95% nonparametric confidence interval). Similarly, in the right hemisphere, the range for the median distance between the two sources was 22.8–49.6 points. Specific comparisons between the individual components of the coordinate system revealed that the Z coordinate (from back to front) differed the most between M50 and M70 components in the left and right hemispheres; however, statistical analysis of the Z coordinate alone did not reach statistical significance (p = 0.086 and p = 0.079, respectively). Figure 4 shows that magnetic source imaging clearly separated the three components in the auditory evoked waveform.

### Developmental Model

Regression analysis, using the least-squares method, revealed that there are many significant developmental patterns that are affected by age that could be accurately modeled. However, in order to be comprehensive, further analysis was performed to probe for possible underlying gender effects. With respect to M70L and M70R latencies, separation by gender increased the significance of the developmental model for females but not males. The M100L amplitude significantly increased in females (R = 0.445, p<0.05); however, this model was not significant in males. Conversely, only in males, significant models were generated based on M100 latency in both the left and right hemispheres (R = 0.486, p<0.01 and R = 0.436, p<0.05, respectively). Because of these unpredictable gender effects, they were not used in constructing our mathematical model. Consequently, the best model for describing developmental changes in children for both males and females was based on the M70 latency. This model also passed the more stringent Bonferroni correction for multiple comparisons (p<0.001). The resulting modeling equations are presented in Table 3.

**Table 3:** The most significant regression modeling equations for auditory evoked responses to binaural tone stimulation.

Component	Regression Equation	Correlates
M70L	81.77-0.896xAge(yrs)*	r^2^ = 0.245
M70R	85.72-1.240xAge(yrs)*	r^2^ = 0.380

* *p<0.001*.

## Discussion

### Morphology age-dependence

The results of the present study demonstrated that the auditory evoked magnetic fields in children were distinctly different from those in adults. M100 was the strongest response in adults; however, M100 could be very small in children. In children, the subcomponents of the middle latency response were clearly separated. Our results showed that in children, M70 might be a stronger and clearer indicator of auditory function than M100. This finding could potentially affect the applications of MEG in research and clinical practice in at least two areas: (1) the M70 response may in fact be the most obvious and robust indicator of auditory representation in the developing brain; (2) it is probably more reliable to use M70 instead of M100 for identifying auditory developmental delays and/or abnormalities.

The majority of the previous research has been performed in adults and focused on M100 [26]–[30]. Additionally, M100 has recently been used in functional mapping for clinical purposes to detect auditory lesions [8]. However, localization of M100 could be troublesome as already shown in previous reports [3]. Oram Cardy and colleagues have found that children have a prominence of M50, however, with the latency at approximately 79.6 ms after stimulus onset [22]. A comparative study of the auditory evoked magnetic fields and intracerebral evoked potentials has revealed a component around 57 ms (Pb1) and another component around 76 ms (Pb2) [6]. Our data showed two components at latencies around 43–50 ms (M50) and 66–77 ms (M70). We consider that the Pb1 and Pb2 described by Oram Cardy's study correspond to the M50 and M70 components in our study. According to the results of the present study with MEG source localization, it is quite probable that M50 and M70 are two distinct components. The separation of the M50 and M70 components could be very important in the search for the most reliable index of auditory development.

### Latency age-dependence

The age-dependence of M100 has been studied extensively, though the age-dependence of either the M50 or M70 complex has rarely been investigated in children. Previous reports have found that the latency of the M100 component in both hemispheres changes with age. EEG studies have also demonstrated that the components of the auditory evoked waveform generally tend to decrease with age [31]. Additionally, previous reports have confirmed that the components of the auditory evoked field shortened with age until children reached 16 years of age, after which they exhibited adult-like waveforms [32]. In the present study, children revealed adult-like waveforms by the age of 17 years old, after which developmental trends were not observed. EEG studies on children, 6–12 years old, focusing on the lateral components of N1_m_ have also confirmed these results [33]. The results of the present study have demonstrated a similar development pattern: the latency of M100 decreases with age. The youngest age group of children had a considerably longer M100 latency than any other age group. This result is also in agreement with previous reports [1], [9], [34], [35].

The age-dependence of M70 was examined in the present study. Previous studies have shown that the latency of a middle latency response using MEG in response to a tone stimulus occurs at about 70 ms and shortens with age [22], [23]. It is highly likely that the component identified at approximately 70 ms corresponds to our M70. Additionally, the present study expands on these preliminary findings by providing a larger database of children, allowing for detailed analysis and comparisons. The present study determined that the latency of the M70 peak was the more localized and more likely the compact source region of auditory development in healthy children. Not only did the latency accurately account for age-related changes in both hemispheres, but it also showed considerable differences between the age groups selected (6–9 years old, 10–13 years old, and 14–17 years old). These results highlight the potential for M70 to become an accurate indicator of auditory injury in abnormally developing children. Finally, the characterization of M50 and its age-dependency in children using MEG was considered a unique finding in the present study. The M50 peak latency in the right hemisphere also correlated well with age, although hardly detectable in adults. Therefore, further analysis of this peak may support the pediatric clinical utility of the M50 response peak.

In addition to M100, the latency of the M50 and M70 in the right hemisphere responses also tended to shorten faster with age than responses in the left hemisphere. These results could indicate a lateralization of development in the brain. A study of EEG coherence and phase found that different development patterns exist in the right and left hemispheres [36]. Our results may provide additional evidence of this age-dependent developmental asymmetry.

The maturational changes in the auditory systems of children illustrate the development of the underlying neurophysiological mechanisms [37]. For example, comparative studies of the long-latency potentials in children with language impairments have linked shorter latencies with an increased transmission velocity in neural networks. Additional EEG testing has shown that age-related changes in latency of auditory evoked potential components gradually decrease over time [38]. In the present study, the latencies of the M50R, M70 and M100 components gradually decreased in children, however, no clear developmental trends were observed for adults. Therefore, because latency changes are based on axon myelination and synaptic density [39], we can infer that this process is evolving in the developing brain, but fairly stable once the brain matures.

### Amplitude age-dependence

The amplitudes of the M70 and M100 changed with age; however, the amplitude of M50 did not show a significant age-dependent change. It was determined that the amplitude of M100 showed a positive linear correlation with chronological age. Increasing amplitudes in the AEF have been correlated with greater auditory synaptic activity in processing a tone stimulus. EEG analysis has also suggested that the largest amplitude in children is seen when children reach 10 years of age [38]. In the present study, the amplitude of the M100 component considerably changed around that age range as well. The results demonstrate that amplitude of the M100 response increases in children as the component becomes more prominent in the auditory evoked waveform and then remains fairly stable in adults.

The amplitudes of M70 did not change linearly with age. The developmental change and the underlying cerebral mechanisms affecting this phenomenon remain unclear. A previously performed MEG study indicated that the amplitude of the response at 70 ms (named M50 in their study but corresponds to our M70) was larger in children than in adults [22]. Interestingly, in the present study, the amplitude of M70 increased with age in young children (6–12 years old) and then began to decrease after the child had reached 13–14 years of age. Since these results were based on 60 children and 20 adults, we consider the non-linear change in the developing brain may well characterize the developmental trajectory of the auditory system. These observations may lay a solid foundation for further study of the developmental delay or abnormalities in various brain disorders.

### Source localization

Previously performed monaural EEG studies on the maturational changes of the auditory evoked potentials have suggested possible differences in the directional orientations of the middle latency components [40]. The present study extended this research by using the superior spatial resolution of MEG in source localization [13]. Importantly, MEG source estimation showed that the M50 and M70 response peaks originated from different subareas of Heschl's gyrus.

### Computational modeling of the developmental change

Given the results from previous reports in combination with our results, we consider that the latency and amplitude of the M50, M70 and M100 provide objective indexes for evaluation of auditory development in the brain. In addition to characterizing these responses in the auditory waveforms, mathematical modeling of the developmental trajectory in children supports the clinical utility of MEG. In the present study, mathematical models were generated for each of the components with respect to hemisphere. The most robust models were based on the latency of the early components: M50R, M70L and M70R. Because the model for M70 proved to be the most consistent and was relatively comparable in both hemispheres, we consider this to be a unique marker of auditory development in children. Furthermore, characterization of these models (shown in Table 3) was also examined to probe for any possible gender effects. Separation by gender improved the significance of our findings in females, but slightly decreased its significance in males. Given the small number of children in each age group, we consider that this might be due to the removal of outliers in the data set; however, none was removed in the data analysis performed in this study. Therefore, a new study with more subjects in this sub-age group may clarify this issue. There is a large variability in both latency and amplitude between different children in each age group. However, this difference is very small when compared to the differences between normal children and children with brain disorders involving in the auditory cortex and/or auditory pathway. Therefore, we consider the present results are scientifically important and clinically useful. Further investigation is warranted to determine the exact effect of gender in the developing auditory system and its lateralization patterns.

These developmental models will be useful for clinical studies as they accurately describe auditory developmental trends in children. Although there is variability within age groups (shown in Figures 2 and 3), the result that much of the variation in the latency of M70 was based solely on the variable “age” is very significant. We are cognizant of the fact that this does pose limitations to the direct clinical application of the results and therefore used a conservative multiple comparisons statistical threshold to highlight the significance of the M70 latency developmental model. The fact that so much of the variation between children can be attributed to one variable is very interesting and informative. Current investigations in our laboratory are working on considering additional variables that would increase the accuracy of our computational developmental model.

In summary, in an effort to characterize the maturational changes in cortical auditory processing, the present investigation recorded evoked auditory responses in children in response to a basic binaural tone. Our results demonstrate that M70 in the right hemisphere was the most sensitive index for identification of the developmental change in children. Using data gathered from adults with the same stimulation paradigm, comparisons across age ranges were performed with respect to each hemisphere. The results confirmed previous studies of the age-specific characteristics of M100. The new findings of the present study include MEG-based separation of the middle latency auditory evoked responses, characterization of their age-dependent developmental differences, and identification of their distinct MEG source estimates. We consider that the results underscore the importance of the early independent M50 and M70 responses as markers in the auditory profile of typical developmental patterns in healthy children. These findings provide a normative database in children against which auditory deficits, functional impairments or other injuries can be better diagnosed.

## Materials and Methods

Sixty healthy normal children (age: 6–17 years, mean age: 11; 30 female and 30 male) and twenty healthy normal adults (age: 19–49 years, mean age: 30 years; 10 female and 10 male) were studied with MEG. These children were recruited from the surrounding Cincinnati area. Since these children were developmentally normal and had no irregular findings reported from MRI, we considered that they were representative of the normal population. According to our previous experience, children under 6 years old were unable to keep still for 30 minutes. In addition, because the purpose of this study was to characterize the development of healthy children, children with speech or language disabilities were excluded from this particular study. The children were divided into three groups, with 20 children in each group: 6–9 years old, 10–13 years old, and 14–17 years old. A written informed consent, at Cincinnati Children's Hospital Medical Center (CCHMC), was obtained from each adult subject or from the parent/legal guardian of each child. This study was approved by Institutional Review Board (IRB) at our medical center. Each subject answered a questionnaire based on the Edinburgh Handedness Inventory [41]. Inclusion criteria for participation were: (1) healthy, without history of neurological disorder, psychiatric disease, or brain injury; (2) normal hearing, vision, and hand movement; (3) current age between 6 years–50 years old; (4) Handedness evaluation was obtained. Exclusion criteria for participation: (1) subject could not keep still; (2) subjects with learning and/or speech/language disability; (3) subjects with unidentifiable magnetic noise; (4) subjects with claustrophobic tendencies or pregnancy were also not used as subjects for MRI purposes.

MEG recordings for this paradigm for a cooperative subject lasted for approximately 30 minutes. Therefore, this study focused on children aged 6–17 years. MEG data can be best recorded from subjects who do not have an implant, such as a cochlear implant device, a pacemaker, or other type of neurostimulator. These devices generate a visible amount of magnetic noise in the acquired MEG data.

### Stimulus

The auditory stimuli were 500 Hz square-wave tones of 200 millisecond duration and a rise and fall time of 10 milliseconds. The tones were generated with BrainX software and presented binaurally with a randomized interstimulus interval of 50±50 milliseconds (0–100 milliseconds) [42]. The stimulation time window totaled 600 milliseconds: 400 milliseconds+interstimulus interval (0–100 m milliseconds). The data acquisition time windows were 400 milliseconds before the trigger (baseline) and 600 milliseconds after the trigger (response time window). We designed this stimulation paradigm for two kinds of data analysis: conventional data averaging and beamformer source estimation, which needed a pre-trigger baseline. The sound was presented at 70 dB nHL through ER-3A ear inserts. The time delay between the sound presented to the subjects and the trigger sent to the MEG system was less than 1 millisecond.

### Data Acquisition

The MEG signals were recorded in a magnetically shielded room (MSR) using a whole head CTF 275-Channel MEG system (VSM MedTech Systems Inc., Coquitlam, BC, Canada) in the MEG Center at CCHMC. Before data acquisition commenced, three electromagnetic coils were attached to the nasion, left and right pre-auricular points of each subject. These three coils were subsequently activated at different frequencies for measuring each subject's head position relative to the MEG sensors. Each subject laid comfortably in the supine position, his or her arms resting on either side, during the entire procedure. The sampling rate of the MEG recording was 6000 Hz per channel. (This high sampling rate was used to allow for high-frequency analysis, which is currently being performed in our laboratory for another study. This study focused on the magnetic signals in the 4–60 Hz band.) The data was recorded with a noise cancellation of third order gradients and without on-line filtering. One hundred trials of binaural presentation were recorded for each subject. Subjects were asked to keep still. If the head movement during one recording was beyond 5 mm, the dataset would be indicated as bad and an additional trial would be recorded.

Three-dimensional Magnetization-Prepared Rapid Acquisition Gradient Echo (MP_RAGE) sequences were obtained for all subjects with a 3T scanner (Siemens Medical Solutions, Malvern, PA). Three fiduciary points were placed in identical locations to the positions of the three coils used in the MEG recordings, with the aid of digital photographs, to allow for an accurate co-registration of the two data sets. All anatomical landmarks digitized in the MEG study were made identifiable in the magnetic resonance images (MRI).

### Data Analysis

For analysis of the time windows of interest, MEG data were averaged for identification of evoked magnetic responses after DC offset correction based on the pre-trigger and with a high pass filter of 4 Hz and low pass filter of 60 Hz. These filtering parameters allowed for clear separation of the M50 and M70 responses. The latency and peak amplitude for each recognizable component in the averaged evoked magnetic response was measured with CTF DataEditor (VSM MedTech Systems Inc., Coquitlam, BC, Canada). A 3D-head model was created with each subject's MRI. Magnetic sources were also volumetrically estimated using MEG Processor, which implemented wavelet based beamformer algorithms [14]. The beamformer algorithm was improved for detecting correlated sources. MEG results were co-registered to the MRI data using three complementary fiducial markers with the Magnetic Source Locator (MSL) [14].

### Statistics

Statistical comparisons between groups of children across pediatric age ranges were performed with the two-sample Student's t-test. Pearson correlation coefficients were computed for the latency and amplitude of M50, M70 and M100 components on the physical sensor waveforms. Developmental changes were characterized by using linear least squares regression analysis for both the children and the adult group data. The threshold of statistical significance for differences was set at p<0.05. For each comparison between two age groups there were twelve measures being tested. Therefore a Bonferroni multiple comparisons correction was applied the data to account for these multiple tests. Thus, if multiple testing is to be taken into account then the significance level for any one of these tests must be reduced from 0.05 to p<0.004 (0.05÷12). Thus, any p-value reported less than 0.004 passed a more stringent criterion for significance and is therefore more noteworthy.

MEG source estimation statistical analysis consisted of computing the distance between the sources. The Euclidean distance between two points (X_1_, Y_1_, Z_1_) and (X_2_, Y_2_, Z_2_) is 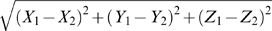. In order to be conservative we determined that each coordinate could have as much as 0.25 units of noise associated with it. For example, the difference between two x-coordinates could be as much as 0.5 units closer than the raw difference would indicate. As a result, the following conservative distance measure using this adjustment for each of the three coordinates was employed: 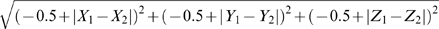.

Statistical analyses were also performed using nonparametric procedures, the Wilcoxon Signed Rank test and Spearman's correlation coefficient as well as robust regression analysis (which does not use least squares solutions). These techniques were used because they require fewer assumptions (e.g., an underlying Gaussian, or Normal, distribution for the data) than the traditional procedures (e.g., Student's t t-test, Pearson correlation, and least squares regression). However, since the results were essentially the same from both types of analyses, we reported the results based on the more traditional procedures. Statistical analyses were performed using SPSS version 15.0 for Windows Evaluation (SPSS Inc., Chicago, IL, USA) and SAS version 9.1 for Windows (SAS Institute Inc, Cary, NC, USA).
